# Inclusion body myositis: from genetics to clinical trials

**DOI:** 10.1007/s00415-022-11459-3

**Published:** 2022-11-18

**Authors:** Sara Nagy, Alaa Khan, Pedro M. Machado, Henry Houlden

**Affiliations:** 1 Department of Neuromuscular Diseases, UCL Queen Square Institute of Neurology, University College London, London, UK; 2 Department of Neurology, University Hospital Basel, University of Basel, Basel, Switzerland; 3 Molecular Diagnostic Unit, Clinical Laboratory Department, King Abdullah Medical City in Makkah, Mecca, Saudi Arabia; 4 Division of Medicine, Centre for Rheumatology, University College London, London, UK

**Keywords:** Sporadic inclusion body myositis, Hereditary inclusion body myositis, Genetic susceptibility, Inflammation, Neurodegeneration, Clinical trials, Gene therapy

## Abstract

Inclusion body myositis (IBM) belongs to the group of idiopathic inflammatory myopathies and is characterized by a slowly progressive disease course with asymmetric muscle weakness of predominantly the finger flexors and knee extensors. The disease leads to severe disability and most patients lose ambulation due to lack of curative or disease-modifying treatment options. Despite some genes reported to be associated with hereditary IBM (a distinct group of conditions), data on the genetic susceptibility of sporadic IBM are very limited. This review gives an overview of the disease and focuses on the current genetic knowledge and potential therapeutic implications.

## Introduction

Idiopathic inflammatory myopathies (IIM) are mostly autoimmune muscle diseases characterized by muscle inflammation, weakness, and a chronic disease course. Inclusion body myositis (IBM) is the most common acquired myopathy above 50 years of age with a variable prevalence of 50–180 per million in this age group, depending on geography and ethnicity [1–3]. However, these numbers are thought to be underestimations in particularly due to misdiagnosis.

IBM is more common in males (2:1) and manifests at a mean age of 60 years [4]. Its phenotype is distinct from other IIMs regarding the insidious onset (per definition duration > 6–12 months) and the clinical pattern. Typically, patients present with a painless and predominantly asymmetric weakness of the knee extensors and long finger flexors, however, both distal and proximal muscle groups become affected, and somewhat less commonly, also the facial and bulbar muscles [4]. Frequent falls are reported, and the slow decline of 4–28% per year leads to loss of ambulation within 10–15 years [5, 6]. Premature mortality is mostly related to dysphagia and consequently aspiration pneumonia as the disease progresses [7].

Although clinical characteristics such as dominant weakness of knee extensors over hip flexors and finger/wrist flexors over wrist extensors are suggestive of the disease, phenotypic variation exists. Diagnosis is underpinned by clinical and histological findings (infiltration of intact fibres by predominantly CD8+ T-cells, major histocompatibility complex (MHC) class I upregulation, rimmed vacuoles, presence of protein p62 or TAR DNA-binding protein 43 (TDP-43)-positive inclusions or amyloid (congophilic) inclusions, cytochrome c oxidase (COX)- negative fibres, cytoplasmic and/or intranuclear tubulofilaments, and/or pseudo-neurogenic changes, laboratory data (creatine kinase, autoantibody profile), electromyography and muscle magnetic resonance imaging (MRI) can be used as supportive tools [8–10]. Seropositivity for anti-cytosolic 5′-nucleotidase 1A  (anti-cN1A) autoantibodies seemed to be a promising marker in clinical practice, however, they turned out to be non-specific and widely detectable in other conditions. Moreover, literature data are not consistent on the association of anti-cN1A with prognostic markers and survival, therefore, its significance in the IBM diagnostic workup needs to be further evaluated [11, 12].

In view of the reported diagnostic delay of 5–6 years in average in IBM [4], more disease awareness is urgently needed to promote early diagnosis making. Most common misdiagnoses include motor neuron disease, other inflammatory myopathies such as polymyositis, dermatomyositis and immune-mediated necrotizing myopathies, and muscular dystrophies which can also exhibit rimmed vacuoles and signs of inflammation on muscle biopsy [13].

**Fig. 1:**
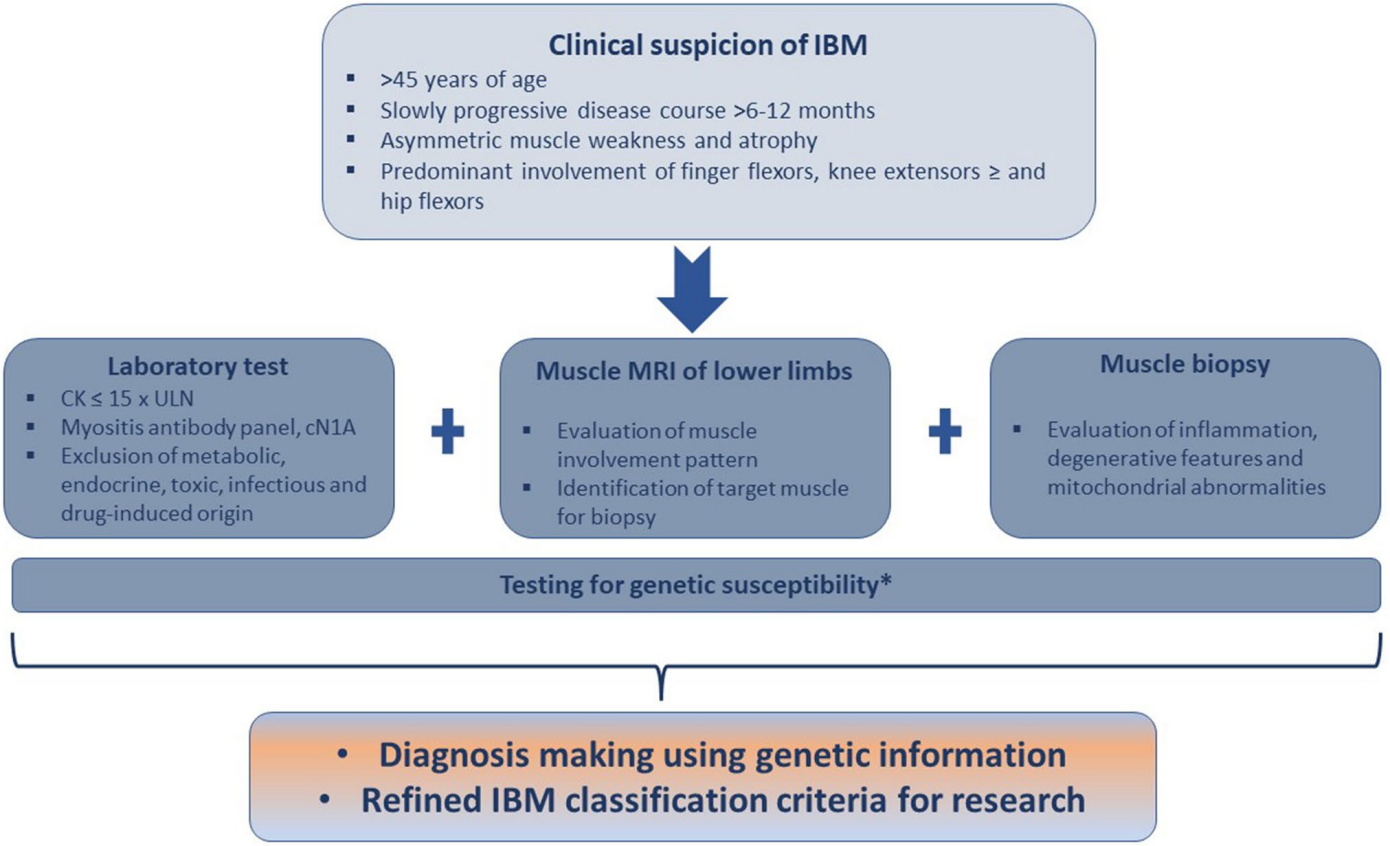
IBM diagnostic workup and improved (more sensitive and specific) classification criteria with *future inclusion of testing for genetic susceptibility

In the hope of increasing therapeutic possibilities in rare diseases such as IBM, early detection of affected patients is as essential for clinical trials as it is for clinical practice. Genetic investigations do not only contribute to the better understanding of disease pathomechanism and the development of potential new treatment strategies but can also serve to improve diagnosis making (Fig. 1). Identification of associated single nucleotide polymorphisms (SNP), gene mutations, and haplotype subtypes can define disease-related genetic susceptibility and have the potential to be integrated in updated classification criteria, if specific enough.

This review gives an update on our current genetic knowledge of IBM and highlights some potential implications for clinical practice and research setting.

## Disease pathomechanism

**Fig. 2:**
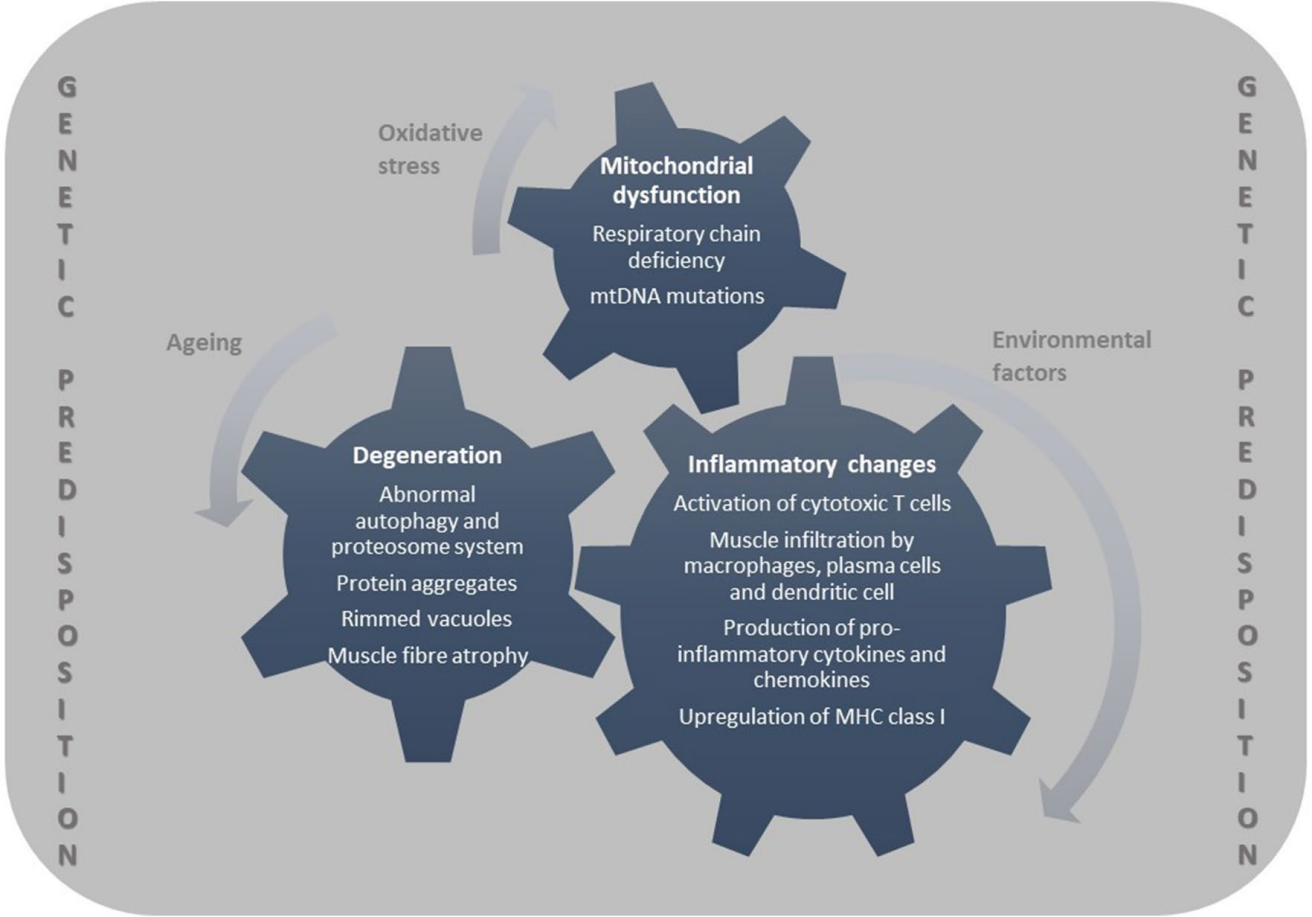
Schematic representation of IBM disease pathomechanism including inflammatory, degenerative, and mitochondrial pathways, and including the role of genetic susceptibility in every underlying mechanism

It is not possible to address genetic factors without talking about the suspected disease pathomechanism first. Evidence supports that IBM is a complex multifactorial disorder, where inflammatory, degenerative, and dysfunctional mitochondrial pathways contribute to disease development. In addition to which, environmental factors and ageing play a role (Fig. 2). IBM is considered as both an autoimmune and neurodegenerative disorder. Histology provided insight into an extensive immune reaction compared with other myopathies, with endomysial infiltration of predominantly CD8+ cytotoxic T lymphocytes, plasma cells, dendritic cells, macrophages, and fibres expressing MHC class I [7]. Signs of ongoing degeneration include the formation of rimmed vacuoles, (β)-amyloid, sarcoplasmic p62, microtubule-associated protein light chain 3 (LC3), valosin containing protein (VCP) and TDP-43, immunoreactive aggregates, myonuclear degeneration and tubulofilaments seen on electron microscope. Mitochondrial dysfunction is associated with the presence of ragged-red fibres and COX-, as well as succinate dehydrogenase (SDH)-positive fibres [7, 14].

The primary trigger that induces a cascade of autoimmune and degenerative responses has not yet been defined, and there is an ongoing debate whether autoimmunity is a primary or secondary phenomenon. However, evolving data suggest that inflammation is the driving factor in IBM disease pathogenesis. This is supported by the similarity with human T-lymphotropic virus type 1 (HTLV-1)-, and human immunodeficiency virus (HIV)-associated polymyositis/IBM complex disease evolution with initially CD8+ T cell-related inflammation followed by subsequent progressive mitochondrial dysfunction and degeneration [7, 15, 16].

According to one of the current hypotheses, a yet unknown chronic inflammatory stimuli induces endomysial infiltration of CD8+ T cells and their clonal expansion into highly differentiated cells with aggressive cytotoxic features, pro-inflammatory cytokine and chemokine releasing properties, and interferon gamma (IFNγ) production [7, 16]. The autoimmune environment further leads to transformation of CD20+ B cells into plasma cells, and consequently, production of autoantibodies [7]. So far, only cN1A could be identified as a target antigen of these antibodies, which can be detected in up to 60% of IBM patients [11, 12]. IFNγ has been shown to induce MHCI upregulation and endoplasmic reticulum (ER) stress, and to promote the cytoplasmic aggregation of proteins such as VCP, p62, LC3 and TDP43. The overloaded degradation system and dysfunctional autophagy and ubiquitin–proteasome system further contributes to the formation of these protein aggravates [8, 16]. An important link between inflammatory and degenerative pathways could be the tumour necrosis factor alpha (TNF-α) induced reduction of those micro-ribonucleic acids (RNAs) which play a role in muscle differentiation (miRNA-1, miRNA-133a, and miRNA-133b) [8].

Mitochondrial dysfunction is seen as a result of the inflammatory cascade [8, 17]. Mitochondrial damage is maintained via pro-inflammatory cytokines such as interleukin (IL)1-β and TNF-α, by the production of reactive oxygen and nitrogen species as well as ER stress. The oxidative stress induced by cytokines leads to increased mitochondrial membrane permeability and uncontrolled transport of substances [17]. Consequently, a dysfunctional respiratory chain follows and the accumulation of mitochondrial DNA (mtDNA) mutations [8]. Damaged and dysfunctional mitochondria are not sufficiently eliminated in IBM through the autophagy process called mitophagy, which further accelerates mitochondrial damage.

The severity of mitochondrial damage is reported to be strongly associated with the degree of inflammation and muscle fibre atrophy [17].

## Genetic contributors of sIBM

**Fig. 3:**
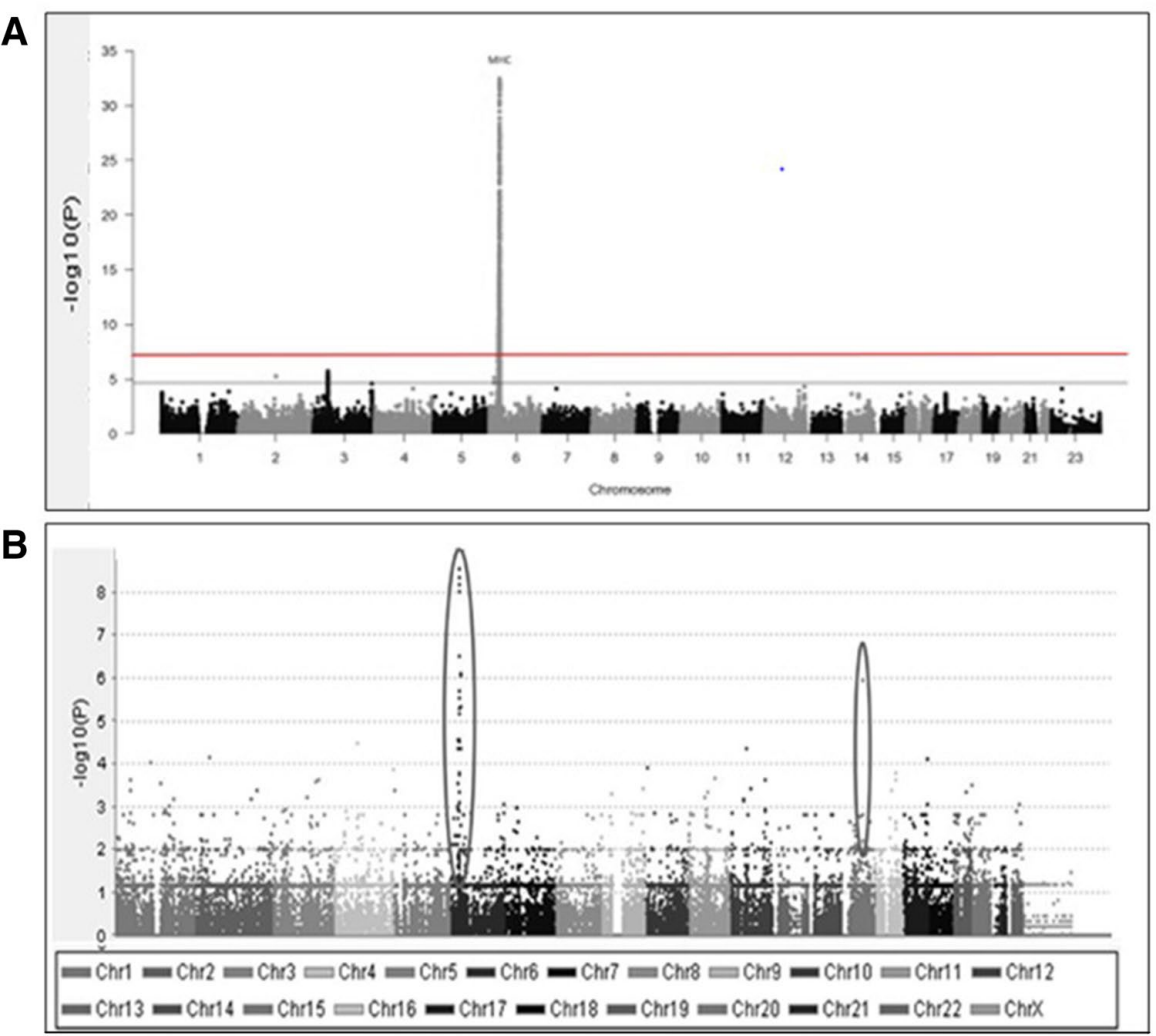
(**A**) Manhattan plot from immune SNP-genotyping based association analysis showing MHC region as the most strongly associated region in sIBM, adapted from [18]. (**B**) Manhattan plot generated from exome-sequencing based association analysis (unpublished data)

Although sporadic IBM (sIBM) is not an inherited Mendelian disease, several genetic risk factors have been shown to play a crucial role in its pathogenesis. Studies confirmed the human leukocyte antigen (HLA) region as the most strongly associated region in sIBM (Fig. 3).

Genetic studies in IIM were conducted by the International Myositis Genetics Consortium (MYOGEN) using the Immunochip genotyping array (Illumina, USA) [18, 19]. Immunochip was designed based on genetic variations associated with autoimmune or inflammatory diseases, with broad coverage across the MHC. These studies were conducted on 2566 IIM cases of European ancestry and identified a strong signal that reached the genome-wide significant threshold of p < 5 × 10−8 in the MHC region. The association analysis in sIBM identified a strong association with HLA-DRB1*03:01, and independent associations with HLA-DRB1*01:01 and HLA − DRB1*13:01; these latter two alleles were specific to sIBM [16]. HLA-DRB1*03:01 and HLA-DRB1*01:01 homozygotes had a lower risk of sIBM than heterozygotes, with the HLA-DRB1*03:01-HLADRB1*01:01 and HLA-DRB1*03:01-HLA-DRB1*13:01 genotypes occurring at a higher frequency than expected. The association analysis of 252 patients with sIBM identified a strong association to chromosome 3 p21.31, implicating a frameshift mutation (rs333) in the C–C chemokine receptor type 5 (*CCR5)* gene as the possible functional variant [16]. CCR5 binds pro-inflammatory chemokines, and a non-functional CCR5 receptor or reduced expression of CCR5 may inhibit T cell migration into muscle fibres. Interestingly, no strong HLA association could be found for the development of anti-cN1A antibodies.

Whole-genome sequencing (WGS) of mtDNA revealed an increased prevalence of somatic large mtDNA deletions and duplications associated with a higher heteroplasmy level in sIBM muscle than controls [20]. There was also an increase in the number of mtDNA somatic protein coding variants and a reduction in the mtDNA copy number variations [20]. Earlier studies have shown that large-scale mtDNA deletions in COX-deficient muscle fibres in sIBM are associated with T-lymphocyte infiltration and muscle fibre atrophy [21, 22]. These results show that mitochondrial muscle ageing is happening faster in sIBM, which may be related to chronic inflammation. Furthermore, IBM patients carrying a very long poly-T repeat allele of the mitochondrial protein translocase of outer mitochondrial membrane 40 (*TOMM40*) had a later age of disease onset [23].

The fact that many pathologically and clinically sIBM-like muscle diseases are associated with known genetic defects supports the idea of contribution of rare variations to sIBM. Rare missense variants could indeed be identified in the FYVE and coiled-coil domain autophagy adaptor 1 (*FYCO1)* gene among sIBM patients via next generation sequencing [24]. Similarly, by analysing candidate genes involved in neurodegenerative diseases including those encoding proteins overrepresented in rimmed vacuoles, rare missense variants in the *VCP* and sequestosome 1 (*SQSTM1)* could be confirmed using whole-exome sequencing (WES) data of IBM patients [25–27]. These findings indicate that impairment of the protein homeostasis, autophagy and the proteasomal degradation plays a role in the pathogenesis of sIBM. However, a small Finnish study could not replicate these results, but found associations in StAR related lipid transfer domain containing 3 (*STARD3*), sphingosine-1-phosphate lyase 1 (*SGPL1*) and SET domain-containing protein 4 (*SETD4*) [28]. Nevertheless, these rare variants cannot be solely responsible for the disease pathogenesis. Furthermore, rare variants association analysis on candidate genes (including amyloid β precursor protein, microtubule-associated protein tau, a-1-antichymotrypsin (AACT), prion protein and C9orf72) associated with neurodegenerative disorders such as amyotrophic lateral sclerosis, frontotemporal dementia, Alzheimer’s disease, Parkinson’s disease, and prion disease did not reach significance [27–29]. Other predisposing variants will probably be identified through WES and WGS in larger sample sizes.

Genome-wide association studies (GWAS) have significantly contributed to our understanding of the pathophysiology of complex non-Mendelian disorders [30], including IIMs. The major limitation of GWAS, particularly in rare diseases like IBM, is the need for large numbers of well-defined cases. Therefore, as part of the Neuromuscular Study Group (NMSG) IBM Genetics Consortium, we performed SNPs-based genotyping array (GSA.v3 genotyping array, Illumina, USA) to conduct the first GWAS study. Our study, currently with 1300 recruited sIBM cases, is the largest sIBM cohort to date. We replicated the previously described association of sIBM with HLA on chromosome 6 (Fig. 3); in addition, we identified new associations with genes involved in immune or neurodegenerative pathways (unpublished data) confirming the role of both inflammatory and neurodegenerative pathways in sIBM pathogenesis. However, experimental investigation is needed to understand these results. We expect to identify several sIBM genetic variants and locus that cluster in disease associated genes. Our GWAS findings will be compared with other ethnic groups including Japanese and European Finnish populations. In the future, RNA sequencing in muscle biopsy will also help to identify the transcriptomic profiles and expression quantitative trait loci (eQTL) in sIBM. The application of RNA sequencing will also allow us to study and dissect splicing abnormalities which may not be detected by exon arrays.

Gender bias is observed in other IIM, with females more typically affected. This is different from sIBM, which has a male predominance. The reason for this difference has not yet been identified, however, hypotheses suggest that there is a gene dosage effect of genes escaping X-inactivation in 46, XX females when compared to 46, XY males. An increased rate of X chromosome aneuploidies was seen in patients with systemic lupus erythematosus and Sjögren's syndrome with a higher rate of 47, XXY (Klinefelter’s syndrome) and 47, XXX [31–33]. The frequency of X chromosome aneuploidies was further investigated in genotyping data generated from an immunotype array. This has identified an elevated rate of 47, XXY in males with polymyositis or dermatomyositis; moreover, the prevalence of 47, XXY males and 47, XXX females were particularly high in sIBM [34]. These findings of an increased rate of X chromosome aneuploidies in IIM may suggest a mechanistic insight in a subset of patients and in IIM more generally.

## What we learned from the genetics of familial IBM

There have been reports of a familial distribution of IBM (fIBM), supporting the role of genetic factors in the disease pathogenesis [35]. At least two siblings were affected in these families, but in some cases an autosomal dominant pattern could be seen. Patients presented with the typical phenotype and histology of IBM. HLA phenotypes were analysed in a few cases and an association with the HLA class II DRB1*0301/0302 was found [35]. These findings underline a similar genetic susceptibility in sIBM and fIBM.

## What we learned from the genetics of hereditary IBM

Hereditary IBM (hIBM) is a heterogenous group of disorders with either autosomal recessive or dominant inheritance (Table 1). Patients with hIBM have an earlier disease-onset, and a variable phenotype generally distinct from that of sIBM. The “IBM” designation can be misleading. Muscle pathology lacks signs of inflammation but shows rimmed vacuoles and tubulofilamentous inclusions [36].

**Table 1:**
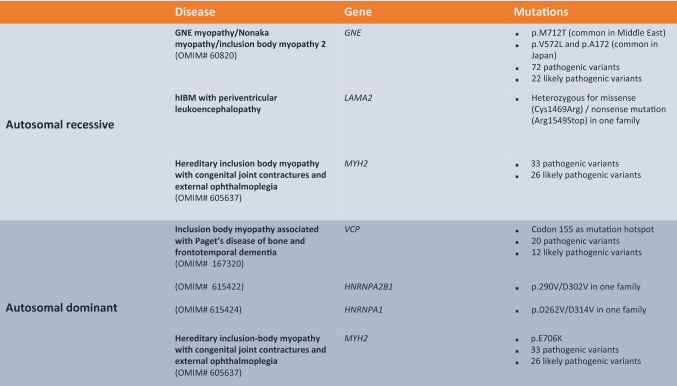
List of known autosomal recessive or dominant hereditary IBM subtypes with genes and common mutations identified Modified from [27]

GNE myopathy, also known as Nonaka myopathy or IBM 2, is a recessive disorder and a result of mutations in the *GNE* gene that encodes the bifunctional UDP-N-acetylglucosamine-2-epimerase/N-acetylmannosamine kinase on chromosome 9p13. The enzyme is part of the sialic acid biosynthetic pathway involved in several important biologic processes including cellular homeostasis, cell adhesion and signal transduction. In Middle Eastern families, a founder mutation in the homozygous state could be identified (p.M712T), while in the Japanese, the homozygous missense mutation p.V572 was common [37–39]. Most other ethnic groups were found to be compound heterozygous for mutations in various regions. Until now, more than 70 pathogenic variants in GNE myopathy have been described. Almost exclusively, inflammation signs are missing in the histology. Affected patients have an earlier disease onset before 45 years, proximal and distal muscle weakness with early involvement of the tibialis anterior and hamstring muscles, and a remarkable sparing of the quadriceps even at later stages. As a result, patients remain ambulatory until very late in the disease course. However, disease severity varies among patients raising the possibility that mutations differ in functional relevance [38].

A few hIBM cases have been described with recessive inheritance, and an early-onset quadriceps-involving phenotype with periventricular leukencephalopathy. In one of the families, a new mutation in laminin subunit alpha 2 (*LAMA2)* resulting in a partial laminin α2 chain deficiency was reported [40]. The gene is otherwise also linked to the development of congenital merosin-deficient muscular dystrophy and autosomal recessive limb-girdle muscle dystrophy 23 (LGMDR23). To the most important LAMA-related pathways belong the extracellular matrix organization and the Phosphatidylinositol 3-kinase-protein kinase B (PI3K-Akt) signalling pathway.

Autosomal dominant forms include hIBM in association with Paget's disease of bone and frontotemporal dementia due to mutations in the *VCP* gene. Most patients have muscle involvement, while only half of them manifest bone lesions and about a third dementia [39]. Muscle symptoms at disease onset are variable but mainly include weakness with a limb-girdle distribution and scapular winging. The *VCP* encoded enzyme is a member of the ATPases associated with a variety of activities (AAA-ATPase) and plays an important role in the ubiquitin–proteasome–system among other intracellular processes. Codon 155 seems to be a mutation hotspot [39]. Until now, 20 pathogenic variants have been reported with suspected pathogenicity through impaired protein turnover.

One family with myopathy, Paget’s disease of bone, dementia and ALS was described with a mutation in the heterogeneous nuclear ribonucleoprotein A2/B1 (*HNRNPA2B1*) which encodes HNRNPA2 and HNRNPB1 proteins that play a role in the packaging of nascent mRNA, thus, in several mRNA-related processes [41]. Another family with myopathy and Paget’s disease of bone had a mutation in *HNRNPA1*, associated with mRNA metabolism and transport [41].

HIBM with congenital joint contractures, ophthalmoplegia and rimmed vacuoles is a slowly or non-progressive disease with mild myopathy linked to a mutation in the myosin heavy chain IIa (*MYH2*) gene that encodes a protein that functions in skeletal muscle contraction. Both autosomal dominant and recessive inheritance have been described [42].

Some other myopathies with histological features of inclusion bodies have been moved to other groups of myopathies. An autosomal dominant or recessive myopathy (OMIM# 601419) with weakness in the distal lower limbs and the quadriceps due to desmin mutations belongs now to the group of myofibrillary myopathies, similarly to the autosomal dominant hereditary myopathy with early respiratory failure (OMIM# 607569) caused by a mutation in titin (TTN). Welander’s distal myopathy (OMIM# 604454) due to autosomal dominant or recessive mutation in the Tia1 cytotoxic granule-associated RNA binding protein (*TIA1)* gene causes weakness in the distal long extensors of the hand and feet [36, 39].

## Clinical trials and putting genetic knowledge into practice

Evidence shows that IBM is refractory to immunosuppressive and immunomodulatory treatments, questioning the primarily autoimmune origin of the disease. One hypothesis, however, suggests that the lack in treatment response could potentially be explained by escape mechanisms of highly differentiated CD4+ and CD8+ T cells. During differentiation, cytotoxic cells lose CD28 positivity, but gain CD244, CD57, and killer-cell lectin like receptor G1 (KLRG1) expression helping them to avoid lymphocyte apoptosis and lymphocyte activation-induced cell death. The phenomenon is also seen in highly differentiated clonal T cells from the T cell large granular lymphocytic leukaemia (T-LGLL) population [7].

Furthermore, the diagnostic delay in IBM leads to treatment initiation at more advanced disease stages, already marked by degeneration, fiber atrophy and fibrosis. At this stage, anti-inflammatory effects may be insufficient. However, recent experiments using a xenograft model by transplanting human IBM muscle into the hindlimb of immunodeficient mice, suggested that T cell depletion does not alter muscle degenerative pathology in IBM; indeed, the authors observed that reduction of human T cells within IBM xenografts by treating mice intraperitoneally with anti-CD3 (OKT3) suppressed MHC-I up-regulation, but rimmed vacuoles and loss of TDP-43 function persisted [43].

Large, prolonged, double-blind, and placebo-controlled trials are scarce in IBM. In clinical practice, there is a lack of improvement with glucocorticoids, although no large trials have been conducted to test efficacy. While azathioprine lacks evidence of effectiveness, open label trials using alemtuzumab or with antithymocyte globulin (ATG) in combination with methotrexate suggested slowing in disease progression, however, placebo controls were not included [44–46]. Intravenous immunoglobulins were suggested as potentially influencing disease progression and dysphagia [47], however, larger, and longer studies are needed, and this treatment effect remains to be proven. A small pilot study with oxandrolone did not meet the primary endpoint of whole-body maximum voluntary isometric contraction testing (MVICT), and most of the secondary endpoints were also negative [48]. Other biological treatments such as INF and anti-TNF agents did not show clear benefits [44]. Arimoclomol, an investigational drug that amplifies heat shock protein response, was also ineffective. Ongoing clinical trials in IBM include a phase 3 trial with sirolimus (rapamycin), which is known to block the activity of T effector cells while preserving T regulatory cells, further, to induce autophagy (NCT04789070). A phase 1 trial is currently evaluating the safety of stem cell injections in the muscle (NCT04975841), while another phase 1 trial is being conducted using the investigational drug ABC008, a humanized monoclonal antibody (mAb) specific for KLRG1 (NCT04659031) [49]. Treatment trials aiming to correct dysfunctional mitochondrial pathway, or to interrogate with autophagy, might follow in the upcoming years.

In view of the increasing evidence of genetic susceptibility in IBM, the potential of gene therapy should also receive attention. The success of gene therapy in spinal muscular atrophy (SMA), a hereditary motor neuron disease, was a breakthrough which rapidly led to increasing efforts in investigating genetic treatments in other degenerative diseases as well, including IBM. In the recent years, the myostatin signalling pathway, which was reported to be upregulated in IBM, received special interest. Myostatin belongs to the tumour growth factor beta (TGF-β) family and is a negative regulator of skeletal muscle mass. A phase 1/2a trial using adeno-associated virus (AAV) delivery of follistatin, a natural inhibitor of the myostatin receptor, showed improvement in the 6-minute-walk test (6MWT) when injected into the quadriceps muscle, however, only six patients were tested and no control group was included [50, 51]. A trial of the with bimagrumab, a fully human monoclonal antibody that binds competitively to activin type II receptors (ActRII) with greater affinity than the natural ligands activin and myostatin, was negative; Bimagrumab had no beneficial effect on the selected primary endpoint, 6MWD, after 52 weeks of treatment, although a dose-dependent effect on lean body mass was observed with Bimagrumab treatment, confirming its biological activity on skeletal muscle mass [52, 53].

The identification of associations with genes involved in immune or neurodegenerative pathways using WES and WEG could open a new treatment opportunity. Gene-based therapies alone or in combination with other treatments have the potential to influence disease progression in at least a subgroup of patients, if not in all.

## Conclusion

IBM remains a complex and challenging neuromuscular disorder where deeper understanding of disease pathomechanism and the identification of potential genetic contributors represent an unmet need.

Further improvement of current diagnostic criteria and the definition of potential genetic and/or laboratory biomarkers are necessary to improve diagnostic accuracy, reduce diagnostic delay and meet the therapeutic window of opportunity for immunomodulatory drugs at early disease stages. Given the importance of neurodegeneration in the disease development especially at later stages, drug interventions must also aim to inhibit intracellular processes that lead to muscle atrophy and fibrosis. The overlaps with hIBM might help to develop a better understanding of the degenerative cascades of sIBM and to define target molecules. Insights into the underlying pathomechanism of IBM could further be applied in other degenerative diseases of the nervous system, either central or peripheral.

## Abbreviations

6MWT: 6-minute walk test
AAA-ATPase: ATPases associated with various cellular activities
AACT: a-1-antichymotrypsin
AAV: Adeno-associated virus
ActRII: Activin type II receptors
ATG: Antithymocyte globulin
c1NA: Cytosolic 5′-nucleotidase 1A
CCR5: C–C chemokine receptor type 5
COX: Cytochrome c oxidase
DNA: Deoxyribonucleic acid
eQTL: Expression quantitative trait loci
ER: Endoplasmic reticulum
fIBM: Familial inclusion body myositis
FYCO1: FYVE and coiled-coil domain autophagy adaptor 1
GNE: Glucosamine (UDP-N-acetyl)-2-epimerase/N-acetylmannosamine kinase
GWAS: Genome wide association study
hIBM: Hereditary inclusion body myositis
HIV: Human immunodeficiency virus
HLA: Human leukocyte antigen
HNRNPA2B1: Heterogeneous nuclear ribonucleoprotein A2/B1
HTLV-1: Human T-lymphotropic virus type 1
IBM: Inclusion body myositis
IFNγ: Interferon gamma
IIM: Idiopathic inflammatory myopathy
IL1-β: Interleukin 1-β
LAMA2: Laminin subunit alpha 2
LC3: Microtubule-associated protein light chain 3
LGMDR23: Limb girdle muscular dystrophy 23
KLRG1: Killer-cell lectin like receptor G1
mAB: Monoclonal antibody
MHC: Major histocompatibility complex
MRI: Magnetic resonance imaging
mtDNA: Mitochondrial DNA
MYH2: Myosin heavy chain IIa
MYOGEN: International Myositis Genetics Consortium
MVICT: Maximum voluntary isometric contraction testing
NMSG: Neuromuscular Study Group
OKT3: Anti-CD3 monoclonal antibody
PI3K-Akt: Phosphatidylinositol 3-kinase-protein kinase B
RNA: Ribonucleic acid
SDH: Succinate dehydrogenase
SETD4: SET domain-containing protein 4
SGPL1: Sphingosine-1-phosphate lyase 1
sIBM: Sporadic inclusion body myositis
SMA: Spinal muscular atrophy
SNP: Single nucleotide polymorphism
STARD3: StAR related lipid transfer domain containing 3
SQSTM1: Sequestosome 1
TDP-43: TAR DNA-binding protein 43
TGF-β: Tumor growth factor beta
TIA1: Tia1 cytotoxic granule-associated RNA binding protein
T-LGLL: T-cell large granular lymphocytic leukaemia
TNF-α: Tumour necrosis factor alpha
TOMM40: Translocase of outer mitochondrial membrane 40
TTN: Titin
VCP: Valosin containing protein
WGS: Whole genome sequencing
